# Effects of Apigenin and Luteolin on *Myzus persicae* (Hemiptera: Aphididae) Probing Behavior

**DOI:** 10.3390/ijms26094452

**Published:** 2025-05-07

**Authors:** Anna Wróblewska-Kurdyk, Bożena Kordan, Jan Bocianowski, Katarzyna Stec, Beata Gabryś

**Affiliations:** 1 Department of Botany and Ecology, University of Zielona Góra, Szafrana 1, 65-516 Zielona Góra, Poland; a.wroblewska@wnb.uz.zgora.pl (A.W.-K.);; 2 Department of Entomology, Phytopathology and Molecular Diagnostics, University of Warmia and Mazury in Olsztyn, Prawocheńskiego 17, 10-720 Olsztyn, Poland; bozena.kordan@uwm.edu.pl; 3 Department of Mathematical and Statistical Methods, Poznań University of Life Sciences, Wojska Polskiego 28, 60-637 Poznań, Poland; jan.bocianowski@up.poznan.pl

**Keywords:** *Myzus persicae*, antifeedants, flavonoids, biopesticides, plant resistance, structure-activity relationship

## Abstract

Apigenin and luteolin are products of the phenylpropanoid pathway, where apigenin serves as a substrate for the synthesis of luteolin. Apigenin and luteolin are highly bioactive flavones; therefore, in search of prospective biopesticides, the receptiveness of the polyphagous green peach aphid *Myzus persicae* (Sulzer) (Hemiptera: Aphididae) to apigenin and luteolin was studied. The flavones were applied as 0.1% ethanolic solutions to the host plant leaf surface, and aphid probing and feeding activities were monitored using the Electrical Penetration Graph (EPG) technique. The structural difference between apigenin and luteolin, which was the number of hydroxyl groups in the molecule, had an impact on the activity of these flavones. On apigenin-treated plants, the duration of the first probe was three times as short as on the control and five times as short as on the luteolin-treated plants; the duration of the time to the first ingestion phase within the successful probe was shorter than on the control and luteolin-treated plants; the mean duration of xylem sap ingestion bouts and the proportion of xylem phase in all probing activities were the highest; and the duration of salivation before phloem sap ingestion was the longest. Aphids’ response to luteolin-treated plants was less distinct as compared to apigenin-treated plants.

## 1. Introduction

**Figure 1:**
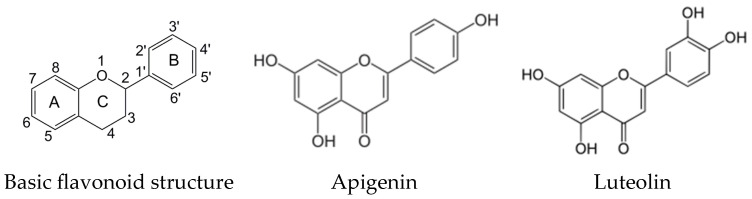
Basic flavonoid structure and chemical structures of apigenin and luteolin.

Apigenin (4′,5,7,-trihydroxyflavone) and luteolin (3′,4′,5,7-tetrahydroxyflavone) (Figure 1) are structurally related natural plant polyphenolic secondary metabolites assigned to the flavone subgroup of flavonoids [1,2].

Flavones, like all flavonoids, are omnipresent in the plant kingdom and can be found in different plant tissues [3,4,5,6]. Flavones, in particular, are an ancient class of plant secondary metabolites: the ability to biosynthesize flavones coincided with the initial colonization of the terrestrial environment by plants [5]. The crucial role of flavones and other flavonoids in plant survival on land is associated with their primary functions as UV protectants and signaling molecules in interactions with other organisms, mainly bacteria and fungi [5]. Their present-day ecological roles also include the participation in plant responses to various abiotic and biotic stresses, and they are involved in multitrophic relationships and other positive and negative interactions between plants and other organisms [6]. The extensive research on the biological significance of flavones has revealed their exceptionally broad bioactivity, which has made them particularly desirable as antioxidant, anti-inflammatory, antitumor, antigenotoxic, antiallergic, neuroprotective, cardioprotective, and antimicrobial agents for application in human medicine [1,2,7].

The involvement of flavones and other flavonoids in the intrinsic mechanisms of plant resistance to pathogens, herbivores, and environmental stresses has led to a growing interest in these allelochemicals for applications in sustainable crop protection practices [5,8,9,10,11,12,13,14,15]. Various ideas have been considered, including the direct application of these compounds as attractants, stimulants, repellents, deterrents, and less-toxic insecticides [13]; indirect application by using agricultural wastes as the flavonoids’ source material [14]; and using flavonoid-releasing plants in “push–pull” systems [15,16]. Flavonoids exuded into the rhizosphere have allelopathic activity [6,10,17] and may prevent infestation by parasitic plants [15,16] and infection by pathogenic fungi [18,19], and they affect the host location of subterranean insects [20]. However, the best-known and best-explored use is the role of flavonoids in plant resistance mechanisms against herbivores in general and phytophagous insects in particular [8]. The breeding, either conventional or through genetic engineering, of herbivore-resistant plant varieties and cultivars is one of the most promising methods of potential sustainable pest management approaches [20,21,22,23,24,25,26]. Therefore, knowledge of individual herbivore species’ strategies for host plant location, recognition, and acceptance is crucial for choosing the correct pest control procedure [27,28].

Insect herbivores exploit plants in a multitude of ways, which determine their dietary specializations [29,30]. Briefly, they differ in the breadth of the host plants’ ranges and in the mode of feeding. In these respects, they can be roughly divided into specialist (mono- and oligophagous) and generalist (polyphagous) herbivores and, on the other hand, depending on their mouthparts’ structure, into chewing–biting and piercing–sucking herbivores [30,31]. The combination of food source preferences and mode of feeding determines the insect herbivore strategy for host plant selection and recognition. In this process, phytophagous insects depend mainly on chemical cues generated by both volatile and non-volatile plant allelochemicals [32]. While volatile cues are used mainly to locate the potential host in biodiverse habitats, non-volatile cues are used at the final stages of host selection, which conclude in either the acceptance or the rejection of the plant source [30,31,32,33,34]. Following the host location in the sequence of events, contact chemoreception is essential for gathering information on the identity and quality of a host plant [28,32]. Chewing–piercing insects are able to recognize their host plants prior to consumption using the external gustatory receptors on their mouthparts, but piercing–sucking insects need to ingest samples of the plant material for gustatory purposes, as their gustatory receptors are located in the cibarium [35].

Aphids (Hemiptera: Aphididae) represent a group of piercing–sucking insect herbivores. The specific mechanism of host plant selection and recognition, i.e., the inevitability of piercing plant tissues and ingesting plant sap for gustatory and nutritional purposes, makes these insects very efficient vectors for plant viruses [36]. It is believed that the indirect damage caused by aphids due to virus transmission exceeds their direct impact on crops [37]. Among aphid plant pests, the green peach aphid, *Myzus persicae* (Sulzer), has an exceptional position: it is extremely polyphagous—its host plant range embraces more than 400 species of 50 plant families, and it is able to transmit more than 100 plant viruses, both persistent and non-persistent [38,39]. In addition, *M. persicae* has evolved diverse mechanisms of resistance to various insecticides [40,41]. Since a global trend in reducing insecticide use has been observed in response to environmental issues, there is a growing demand for the replacement of traditional insecticides, at least in part, by natural product-based insect control agents and/or the introduction of cultivars resistant to aphid infestation [42]. These two optional or complementary approaches involve the use of allelochemicals that may alter the behavior of aphids at various stages of the host plant selection process, essentially at the plant recognition and acceptance phases [42,43,44,45,46].

Flavonoids in general and flavones in particular have great insect-behavior-modifying potential [11,12,13,47,48]. The effects of flavones and other flavonoids on aphid plant selection behavior and plant susceptibility to aphid infestation have been thoroughly studied in recent years. The reported results have demonstrated a significant specificity of flavonoid compound activity toward individual aphid species. As an illustration, quercetin promotes the probing activities of the pea aphid, *Acyrthosiphon pisum* (Harris), within non-phloem and phloem tissues and accelerates access to the phloem in *M. persicae* but has no effect on the probing behavior of the bird cherry-oat aphid, *Rhopalosiphum padi* (L.) [49]; rutin causes a delay in *A. pisum*’s ability to reach sieve elements*,* deters the probing activities of *M. persicae* within non-phloem tissues and does not affect *R. padi* [49]; in *A. pisum,* daidzein causes a delay in reaching phloem vessels and limits sap ingestion, kaempferol causes a reduction in the frequency and duration of the phloem phase, and genistein does not affect aphid probing behavior [50]; hesperidin affects the pre-phloem probing phase in *A. pisum* and the ingestion phase in *R. padi* and does not affect *M. persicae* [51]; naringenin and quercetin have no direct effect on diet ingestion in *A. pisum* [52] but enhance sap ingestion in *M. persicae* [53]. These sample results [49,50,51,52,53] exemplify the very well-known fact that the biological activities of compounds depend on their structural characteristics [53,54,55,56,57].

The present study focused on two structurally related flavonols, apigenin and luteolin. In plant biosynthetic pathways, apigenin is a product of the phenylpropanoid pathway and serves as a substrate for the synthesis of luteolin thanks to the action of flavonoid 3′-hydroxylase [1,2,56,57,58,59]. Both apigenin and luteolin are flavonoids with diverse biomedical activities, including antibacterial, anti-inflammatory, anti-cancer, anti-diabetic, and cardioprotective activities [1,2,58,59]. The involvement of apigenin and luteolin in inter-organismal/interspecies relationships is also known. In particular, apigenin is toxic to the Formosan subterranean termite, *Coptotermes formosanus* Shiraki (Isoptera: Rhinotermitidae), and to the larvae of *Culex quinquefasciatus* Say (Diptera: Culicidae) [60,61]; shows antifeedant activity toward adult striped flea beetles, *Phyllotreta striolata* Fabricius (Coleoptera: Chrysomelidae) [62]; and negatively affects metabolism in predatory fish *Channa punctata* (Bloch) (Anabantiformes: Channidae) [63]. Luteolin negatively affects the development of *Spodoptera exigua* (Hübner) larvae (Lepidoptera: Noctuidae) [64]; stimulates the feeding of leaf beetles, *Chrysomela vigintipunctata* (Scopoli) (Coleoptera: Chrysomelidae) and *Plagiodera versicolora* Laicharting (Coleoptera: Chrysomelidae) [65]; and contributes to the antibiotic resistance of centipedegrass *Eremochloa ophiuroides* (Munro) Hack (Poaceae) to the larvae of the fall armyworm, *Spodoptera frugiperda* (J.E. Smith) (Lepidoptera: Noctuidae) [66].

In search of prospective biopesticides, the receptiveness of aphids to apigenin and luteolin in their diet has also been explored. Studies have involved the development and probing behavior of *A. pisum* [50,67,68] and the black bean aphid, *Aphis fabae* Scopoli (Hemiptera: Aphididae) [69]. There are certain unfocused reports that apigenin and luteolin may reduce plant acceptability in *M. persicae* [70], but no precise research exists on the direct impact of the two flavones on this highly polyphagous and globally important [71] pest aphid.

The aim of the present study was to evaluate the probing and feeding deterrent potential of apigenin and luteolin against *M. persicae*. We applied apigenin and luteolin as 0.1% ethanolic solutions to the host plant leaf surface and monitored aphid probing and feeding activities on treated and untreated plants using the Electrical Penetration Graph (EPG) technique. EPG is the only research technique that visualizes aphid stylet movements and related activities within plant tissues, which are otherwise unavailable for direct observation [72,73]. EPG is commonly applied in studies on Hemiptera–plant relationships [67,68,69,72,73].

We hypothesized that (i) apigenin and luteolin impair green peach aphid activities in non-vascular and vascular tissues, and (ii) structural differences between the two chemically related flavones affect their impact on the behavior of *M. persicae*.

## 2. Results

**Figure 2:**
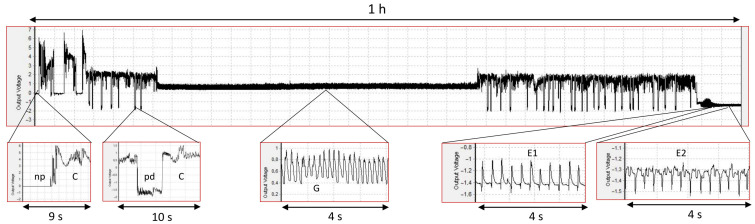
Electrical Penetration Graph recording of *Myzus persicae* stylet activities in plant tissues of *Brassica rapa* ssp. *Pekinensis* treated with 0.1% apigenin. Upper panel illustrates a 1 h section of the 8 h EPG. Lower panels show the details of individual EPG waveforms corresponding to the display in the upper panel. “np”—no probing; “C”—progressive stylet movements within the apoplast and occasional punctures of cells adjacent to the stylet track represented as potential drops, “pds”; “G”—active uptake of xylem sap; “E1”—egestion of saliva into sieve elements; “E2”—passive ingestion of phloem sap from sieve elements.

The probing activity of *Myzus persicae* on untreated and apigenin- and luteolin-treated *Brassica rapa* ssp. *Pekinensis* was monitored for 8 h continuously. Each of the three treatments was replicated 20 times, and the success rate, i.e., the number of complete 8 h EPG recordings suitable for analysis, was high for all plants: 85%, 95%, and 80%, respectively, for untreated and apigenin- and luteolin-treated plants (Table 1, Table 2, Tables S1 and S2; all the traits had a normal distribution). On all plants, *M. persicae* showed various probing activities that embraced stylet penetration within the epidermis and mesophyll (consisting of typical stylet movements in the apoplast with short intracellular punctures represented by pathway waveform “C” and, sporadically, misguided stylet movements in the apoplast caused by their “derailment”, labeled waveform “F”), xylem vessels (i.e., xylem sap ingestion, “G”), and sieve elements (comprising salivation, “E1”, and phloem sap ingestion, “E2”) (Figure 2).

**Figure 3:**
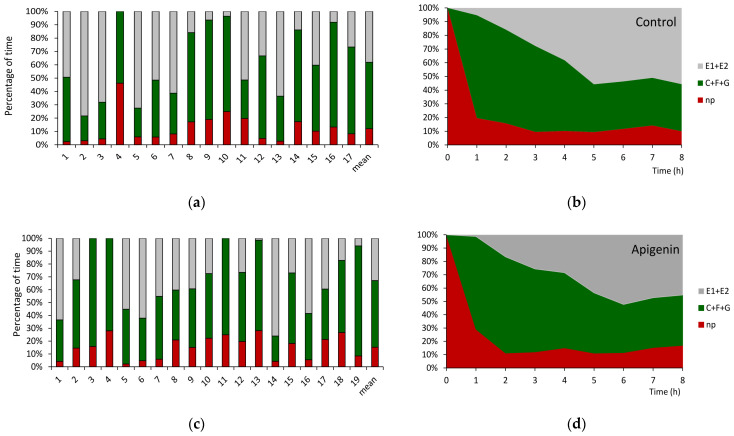

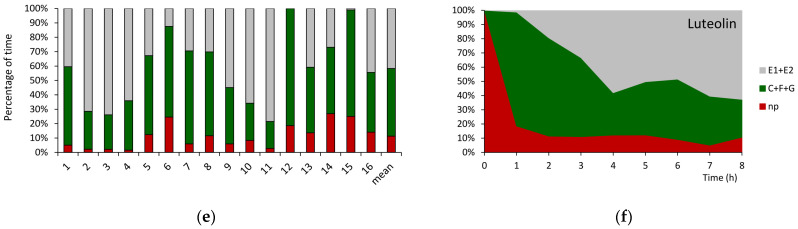
Individual variation and sequential changes in probing behavior of *Myzus persicae* on *Brassica rapa* ssp. *pekinensis* untreated (**a**,**b**) and treated with 0.1% apigenin (**c**,**d**) and 0.1% luteolin (**e**,**f**). Panels (**a**,**c**,**e**) represent the proportion of time (percentage of cumulative time for individual aphids and the mean of the group) devoted to individual probing activities. Panels (**b**,**d**,**f**) represent the proportion of time (average percentage of cumulative time for aphids in the group) devoted to individual activities during the successive hours of the 8 h EPG recording. np = no probing; C + F + G = pathway + derailed stylet activities + xylem phase; E = phloem phase E1 (salivation) + E2 (sap ingestion) activities.

The no-probing activities, i.e., when aphids kept their stylets withdrawn from plant tissues, occupied a similar proportion of the complete experimental time, i.e., 12.5%, 13.1%, and 11.3%, on untreated and apigenin- and luteolin-treated plants, respectively, despite individual variation in behavior between aphids within each treatment (Table 1 and Table S1; Figure 3a,c,e). Correspondingly, the proportions of probing activities spent on pathway (“C”) and phloem activities (“E”) were comparable between the treatments and amounted to, respectively, from 46% of the total probing time on luteolin-treated plants to 53 % on apigenin-treated plants (pathway) and from 42% on apigenin-treated plants to 48 % (phloem sap ingestion) on luteolin-treated plants. Likewise, the frequency and duration of derailed stylet activities in the apoplast (“F”) were similar in all treatments (Table 1 and Table S1). Significant differences occurred in the average duration of xylem sap ingestion, “G”: the longest ingestion periods and the highest proportion of probing time spent on “G” activity occurred in aphids on apigenin-treated plants. The duration of individual bouts of watery salivation, “E1”, preceding the first bout of sustained phloem sap ingestion (“E2 > 10 min”) differed significantly between treatments: the longest “E1” occurred in aphids on apigenin-treated plants (Table 1 and Table S1).

The sequence of aphid activities during the 8 h monitoring period started with the onset of stylet penetration in the epidermis and mesophyll (pathway activities) that followed the initial no probing at the start of the experiment. On control and flavone-treated plants, *M. persicae* began probing within 1–3 min after gaining access to the plants, i.e., after the individual aphids were put on plant leaves to start the EPG recording (Table 2 and Table S2). However, the duration of the first probe was significantly shorter for aphids on apigenin-treated plants as compared to aphids on control and luteolin-treated plants. Nevertheless, the proportion of no-probing activities declined rapidly on all plants, during the first hour of the experiment in favor of pathway activities that predominated in the course of the second and third hours (Figure 3b,d,f). The first phloem phase occurred approximately 3 h after the first stylet penetration event in plant tissues on all plants, and most of that time was occupied by pathway activity; no probing was marginal (Table 2 and Table S2). On control and luteolin-treated plants, the phloem phase was the main activity beginning with the third hour of the experiment onwards with, respectively, 56% and 63% of all activities at the end of the experiment (Figure 3b,f), while on apigenin-treated plants, the phloem phase occupied 45% of all activities at the experiment termination (Figure 3d).

**Figure 4:**
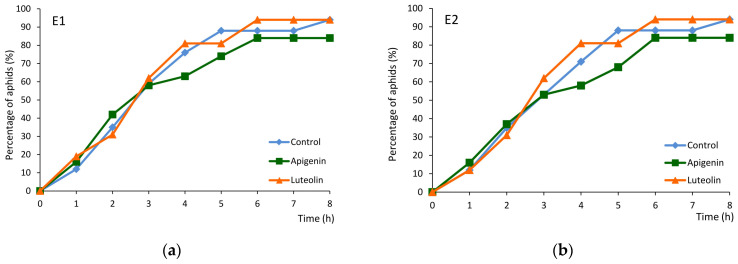
Cumulative percentage of *Myzus persicae* that attained the phloem phase (reached sieve elements) during 8 h EPG monitoring of control untreated *Brassica rapa* ssp. pekinensis and *Brassica rapa* ssp. pekinensis treated trans-epidermally with 0.1% apigenin and 0.1% luteolin. Panel (**a**) represents aphids that showed any contact with sieve elements confirmed by the appearance of the first “E1” waveform (phloem salivation) in the successive hours of the EPG recording. Panel (**b**) represents aphids that showed the first bout of ingestion activity confirmed by the appearance of the first “E2” waveform (phloem sap ingestion) in the successive hours of the EPG recording.

On control and luteolin-treated plants, 94% of aphids reached the phloem phase, while on apigenin-treated plants – 84% (Figure 4a). The elapsed time between the first probe and the first phloem phase, the first sap ingestion phase, and the first sustained ingestion phase was similar for all plants (Table 2 and Table S2). However, within the successful probe, i.e., the probe that included the first bout of phloem sap ingestion, “E2”, the elapsed time from stylet insertion to the onset of ingestion was significantly shorter on the apigenin-treated plants than on the control plants. The first phloem phase was twice as short, on average, on apigenin-treated plants (Table 2 and Table S2).

## 3. Discussion

### 3.1. Effects of Apigenin and Luteolin on Aphid Activities in Non-Vascular and Vascular Tissues

Aphid probing is usually divided into two major phases, probing in non-vascular tissues and probing in vascular tissues, due to the different goals they are to meet. The main destination for aphid stylets is the plant phloem, specifically, the sieve tubes of the phloem, the basic source of nutrients [73,74]. Occasionally, aphids also need to ingest the sap from xylem vessels, principally to minimize the osmotic effects of the consumed phloem sap [75]. To reach the phloem and xylem, which are both vascular tissues embedded within the parenchyma inside plant organs, aphids first need to penetrate the epidermis and then a number of layers of other non-vascular tissues [37,72,73].

Probing within non-vascular tissues, the epidermis and parenchyma (the mesophyll in leaves), involves the penetration of the aphid mouthpart stylets into the apoplast, i.e., within the system of plant cell walls, toward vascular tissues [73]. This activity is mainly the mechanical progressive movement of the stylets, but almost all cells along the stylet pathway are briefly punctured [37,39,73]. These punctures are usually 5–10 s long and include both salivation and ingestion, allowing for the collection of cell content samples used for host recognition [72,73,75]. Probing in non-vascular tissues prior to the first contact with sieve elements is crucial for the acceptance or rejection of a plant: on non-hosts, aphids discontinue probing and withdraw their stylets while still penetrating the mesophyll, and no waveform associated with the phloem phase can be observed in an EPG [76,77]. Similar behavior can be observed in aphids whose host plants are treated with unacceptable xenobiotics: a significant decline in phloem activities can be observed on such plants [78,79,80].

In the present study, the probing behavior of *M. persicae* in non-vascular plant compartments did not differ significantly between treatments, with only two exceptions: the duration of the first probe and the duration of the time to the first ingestion phase within the successful probe. The first probe on the apigenin-treated plants was three times as short as on the control and five times as short as on the luteolin-treated plants. The initial probes on new plants are usually no longer than three minutes and reflect the punctures of epidermal cells [73]. The withdrawal of stylets by *M. persicae* from apigenin-treated leaves soon after the insertion can, therefore, be interpreted as a negative response to this flavone, as was observed in other studies on plant suitability to aphids [79,81]. However, the deterrent effect of apigenin observed here seems short-lived, as neither the general probing activity nor the time and success regarding reaching the phloem were affected. Moreover, the time to reach the sieve element sap ingestion phase within the probe on the apigenin-treated plants was shorter than on the control and luteolin-treated plants.

Probing in vascular tissues is devoted to ingestion. The mechanical movements of stylets pause in favor of the passive or active ingestion of phloem or xylem sap, respectively [72,73,75]. In the present study, two aspects of probing in vascular tissues appeared significant in relation to flavone treatment for plants. First, the mean duration of xylem sap ingestion bouts and the proportion of the xylem phase in all probing activities were highest in aphids on apigenin-treated plants. Second, the duration of watery salivation before the first bout of phloem sap ingestion was longer on apigenin-treated plants as compared to the control. As stated earlier, the ingestion of xylem sap is mainly caused by osmotic stress following the consumption of phloem sap with high osmotic potential [75,82]. However, an increase in xylem sap ingestion has also been observed in other situations, for example, in parasitized aphids [83]; aphids on unsuitable or resistant plants [84,85]; and in relation to aphid age, morph, or developmental stage [85,86]. The increased duration of the xylem phase on apigenin-treated plants found in the present study corresponds to the idea that xylem sap ingestion might limit the negative impact of toxins in the phloem sap by diluting them below their noxious concentration [85]. This possible explanation is also consistent with another finding of our study: increased salivation before phloem sap ingestion on apigenin-treated plants. Generally, salivation into sieve elements precedes any bout of sap ingestion and is presumed to reduce or eliminate the effects of plant defense mechanisms that would have prevented uninterrupted ingestion by aphids [72,73,75]. Therefore, the duration of salivation is considered an indicator of natural plant resistance at the level of phloem vessels and the deterrent effects of exogenously applied xenobiotics, which are also perceptible at the phloem level [73,78,85]. However, the present study showed that despite the initial increase in phloem salivation on apigenin-treated plants, phloem sap ingestion by *M. persicae* was not impeded by any of the flavones applied.

In our previous studies using the same method, we demonstrated that the behavioral response of *M. persicae* and other polyphagous aphid species to flavonoids administered to host plants is rather poor [50,51,53]. The present research provided new evidence for those findings: apigenin had a very limited effect, and luteolin did not affect the probing behavior of *M. persicae* in a significant way. We conducted our experiments using Chinese cabbage as the test plant. Interestingly, “flavones-producing plant species belong to more than 70 different families within the plant kingdom”, but “these compounds seem to be absent in almost all of the about 3000 Brassicaceae species, although many other flavonoid-classes are present, especially flavonols” [5]. However, considering the extremely wide spectrum of host plants for *M. persicae,* reaching far beyond Brassicaceae [38], it is highly probable that the green peach aphid made contact with apigenin and luteolin during coevolutionary processes and developed a high tolerance to these compounds.

### 3.2. Effects of Apigenin and Luteolin Molecular Structures on Aphid Probing Behavior

The structural differences between apigenin and luteolin refer to the number and positions of hydroxyl groups in the molecule. Apigenin has three hydroxyl groups at positions C-5 and C-7 of the A-ring and C-4′ of the B-ring of the basic flavonoid structure, while luteolin has four hydroxyl groups at positions C-5 and C-7 of the A-ring and C-3′ and C-4′ of the B-ring [1,2].

In the search for feeding deterrents against *M. persicae*, a number of structure–activity studies have been carried out (e.g., [80,87,88,89]). These studies show that the natural presence or synthetic incorporation of hydroxyl groups into molecules of various natural compounds can strongly affect their activity. Hydroxylactones derived from natural piperitone cause a significant decrease in probing in non-vascular tissues and the duration of phloem sap ingestion in relation to the original compound [78]. On farnesol-treated plants, the total duration of the phloem phase and the mean duration of individual sustained ingestion periods were significantly lower and the proportion of phloem salivation was higher than on control plants; on nerolidol-treated plants, the occurrence of the first phloem phase was delayed, and the frequency of the phloem phase was lower than on control plants. Both farnesol and nerolidol are natural compounds [80]. *β*-Damascone, a natural terpenoid, appears to be a weak attractant that is close to inactive toward *M. persicae*, but dihydro-β-damascol is a strong deterrent that significantly increases salivation into sieve elements and reduces phloem sap ingestion [88]. Jasmonate derivatives containing a hydroxy group, especially in correlation with a lactone ring, cause a decrease in the duration of non-probing intervals and an increase in the duration of sap ingestion periods as compared to the natural compound cis-jasmone [90].

In the present study, we determined that the structural difference between apigenin and luteolin, which was the number of hydroxyl groups in the molecule, also had an impact on the activity of these flavones. The mean duration and proportion of xylem sap ingestion in total aphid probing on luteolin-treated plants were lower than on apigenin-treated plants. The duration of salivation into sieve elements preceding the first bout of sustained sap ingestion was shorter on luteolin-treated plants than on apigenin-treated plants. The first probe was longer, and the proportion of phloem sap ingestion activity at the termination of the experiment was higher on luteolin-treated plants than on apigenin-treated plants.

## 4. Materials and Methods

### 4.1. Compounds, Insect and Plant Cultures, and Application of Compounds

Apigenin and luteolin were purchased from Sigma–Aldrich (Poznań, Poland).

Aphids (*Myzus persicae*) (kept as a multiclonal colony) and plants (Chinese cabbage *Brassica rapa* ssp. *pekinensis*) were reared in a laboratory at 20 °C, 65% r.h., with a 16:8 (L/D) photoperiod. One- to seven-day-old apterous females of *M. persicae* and three-week-old plants with 4–5 fully developed leaves were used for experiments. All experiments were carried out under the same temperature, relative humidity, and photoperiod conditions. Bioassays were started at 10–11 a.m. To imitate the natural environment under laboratory conditions, apigenin and luteolin were presented to aphids by treating their host plants. Preparation and application of the compounds followed the procedure described by Polonsky et al. [87], later modified by Gabryś et al. [88]. The compounds were applied to one leaf of an intact plant by immersing it in a 0.1% ethanolic solution of a given compound for 30 sec. Control leaves of similar size were immersed in 70% ethanol, which was used as a solvent for apigenin and luteolin. Treated and control leaves were allowed to dry for 1 h before the start of the experiment to permit the evaporation of the solvent.

### 4.2. Behavioral Responses of Aphids During Probing and Feeding: Electrical Penetration Graph Technique

Behavioral responses of aphids during probing and feeding were monitored by recording aphid stylet activities in plant tissues using the Electrical Penetration Graph technique. In this experimental setup, the aphids and plants were made parts of an electrical circuit by attaching them to electrodes (aphid electrode: 25–30 mm long ø18 μm golden wire attached to aphid dorsum with water-soluble silver paint; plant electrode: copper wire inserted into the soil). The electrical circuit was completed when the aphid inserted its stylets into the plant. Weak voltage was supplied in the circuit, and all changing electric properties were recorded as EPG waveforms that could be correlated with aphid activities and stylet positions in plant tissues [72]. The values of parameters derived from EPG recordings—e.g., the duration of probing, duration of phloem sap ingestion, and number of probes—reflect the suitability of a food source to aphids [73,74,75,76,81,89]. After the attachment of the golden wire electrode, aphids were starved for 1 h prior to the experiment. Each aphid was given access to a freshly prepared leaf. A Giga-8 DC EPG system with 1 GΩ of input resistance (EPG Systems, Wageningen, The Netherlands) was used to record EPGs. EPGs were recorded using the Stylet+d_2019 software (EPG Systems). The probing behavior of 20 apterous females per studied flavone/aphid combination was monitored for 8 h continuously. Each aphid was given access to a freshly prepared plant leaf of an intact plant. Each plant–aphid set was considered a replication and was tested only once. The number of replications (EPG recordings) for each plant treatment was 20. Recordings that terminated due to an aphid falling from the plant or where the EPG signal was unclear and the total duration of no probing exceeded 75% of the total recording time were discarded from the analysis. Only the replications that included complete 8 h recordings were kept for analysis.

### 4.3. Identification of EPG Waveforms and Calculation of EPG Variables

Individual EPG waveforms generated by aphids on control and apigenin- and luteolin-treated plants were identified manually using the Stylet+ software (EPG Systems, Wageningen, The Netherlands). The following EPG patterns were distinguished: np (no probing; aphid stylets outside the plant), C (pathway phase; intercellular apoplastic stylet pathway, including some pds), F (the derailed stylet activities), G (xylem sap ingestion), E1 (salivation into sieve elements), and E2 (ingestion of phloem sap). The E1/E2 transition patterns were included in E2. A number of sequential (i.e., describing the sequence of events during the recording) and non-sequential (i.e., referring to frequency and total and average duration of patterns) parameters were calculated and analyzed in configurations related to activities in peripheral and vascular tissues using the MS Excel workbook for automatic calculation [91].

### 4.4. Statistical Analysis

The normality of the distributions of the studied traits was tested using Shapiro–Wilk’s normality test [92]. The homogeneity of variance was verified using Bartlett’s test. One-way analyses of variance (ANOVA) were carried out to determine the effects of the experimental treatment on the variability of the examined traits for each trait independently. The arithmetic means and standard error of the mean (SEM) of traits were calculated. Tukey’s honest significant differences (HSDs) were estimated at a significance level of *α* = 0.05, corrected for multiple testing using the Benjamini–Hochberg method. Homogeneous groups for the analyzed traits were determined based on HSD values. All the analyses were conducted using the GenStat 23 statistical software package [93].

## Figures and Tables

**Table 1:** Probing behavior of *Myzus persicae* on *Brassica rapa* ssp. *Pekinensis* untreated and treated with 0.1% apigenin and 0.1% luteolin: non-sequential EPG parameters.

EPG Variable	Control		Apigenin		Luteolin		HSD_0.05_	*F*-ANOVA
	n ^1^	Mean ± SEM	n ^1^	Mean ± SEM	n ^1^	Mean ± SEM		
No probing								
Total duration of np	17	60.2 a ± 12.9	19	73.4 a ± 9.90	16	54.2 a ± 10.3	31.5	0.454
Number of np	17	41.2 a ± 6.60	19	41.6 a ± 5.10	16	38.8 a ± 5.80	16.5	0.936
Mean duration of np	17	1.40 a ± 0.10	19	1.70 a ± 0.20	16	1.30 a ± 0.20	0.42	0.116
Probing								
Total probing time	17	419.8 a ± 12.9	19	406.6 a ± 9.9	16	425.8 a ± 10.3	31.5	0.454
Number of probes	17	41.1 a ± 6.60	19	41.5 a ± 5.10	16	38.7 a ± 5.70	16.4	0.935
Number of short probes (C < 3 min)	17	26.8 a ± 5.10	19	27.6 a ± 4.20	16	24.3 a ± 4.20	12.8	0.867
Pathway phase								
Total duration of C	17	196.0 a ± 22.7	19	208.0 a ± 19.0	16	190.6 a ± 18.0	56.8	0.817
Number of C	17	43.7 a ± 6.70	19	45.2 a ± 4.80	16	42.0 a ± 6.00	16.5	0.927
Mean duration of C	17	5.70 a ± 0.80	19	5.20 a ± 0.50	16	5.20 a ± 0.40	1.73	0.746
Proportion of probing spent in C (%)	17	49.0 a ± 6.60	19	53.0 a ± 5.70	16	46.2 a ± 5.10	16.6	0.711
Derailed stylet activities								
Total duration of F	17	29.2 a ± 9.80	19	19.0 a ± 8.70	16	18.4 a ± 7.20	24.8	0.623
Number of F	17	0.80 a ± 0.20	19	0.50 a ± 0.20	16	0.40 a ± 0.20	0.57	0.503
Mean duration of F ^2^	9	42.1 a ± 9.20	6	32.9 a ± 9.60	6	45.6 a ± 11.9	19.2	0.376
Proportion of probing spent in F ^2^ (%)	9	12.9 a ± 3.40	6	14.2 a ± 4.60	6	12.2 a ± 3.10	7.07	0.841
Xylem phase								
Total duration of G	17	12.7 a ± 3.90	19	23.6 a ± 6.8	16	16.6 a ± 5.70	16.3	0.393
Number of G	17	0.60 a ± 0.20	19	0.60 a ± 0.16	16	0.70 a ± 0.20	0.53	0.977
Mean duration of G ^2^	8	21.5 b ± 2.00	10	38.5 a ± 7.70	8	26.1 ab ± 7.50	13.6	0.04
Proportion of probing spent in G ^2^ (%)	8	6.60 b ± 1.10	10	11.2 a ± 2.10	8	7.80 ab ± 1.80	3.70	0.044
Phloem phase: general								
Total duration of phloem phase E (E1 + E2)	16	193.2 a ± 30.4	16	185.1 a ± 25.3	15	213.3 a ± 28.7	76.3	0.752
Total duration of E1	16	1.80 a ± 0.30	16	7.40 a ± 4.60	15	2.70 a ± 0.60	7.38	0.252
Total duration of E2	16	191.5 a ± 30.6	16	177.9 a ± 26.2	15	210.9 a ± 28.9	77.4	0.693
Phloem phase: salivation (E1)								
Number of E1	17	1.90 a ± 0.30	19	3.00 a ± 0.60	16	2.90 a ± 0.60	1.53	0.306
Mean duration of E1 ^2^	16	0.90 a ± 0.10	16	2.30 a ± 1.50	15	0.90 a ± 0.70	2.44	0.371
Duration of the E1 followed by 1st E2 ^2^	16	1.70 a ± 0.30	16	2.60 a ± 0.50	15	2.20 a ± 0.40	1.07	0.185
Duration of E1 followed by 1st E2 > 10 min ^2^	15	1.00 b ± 0.10	15	1.80 a ± 0.26	14	1.50 ab ± 0.30	0.60	0.035
Contribution of E1 to phloem phase (%) ^2^	16	3.30 a ± 1.80	16	6.10 a ± 3.60	15	5.80 a ± 4.30	9.08	0.79
Proportion of probing spent in E1 (%)	16	0.40 a ± 0.90	16	1.80 a ± 1.20	15	0.60 a ± 0.10	1.88	0.261
Phloem phase: sap ingestion (E2)								
Number of E2	17	1.80 a ± 0.30	19	2.70 a ± 0.60	16	2.60 a ± 0.60	1.45	0.39
Number of E2 > 10 min	17	1.10 a ± 0.20	19	1.70 a ± 0.30	16	1.70 a ± 0.30	0.82	0.253
Mean duration of E2 ^2^	16	155.3 a ± 35.1	16	85.5 a ± 21.0	15	137.1 a ± 32.0	80.9	0.195
Proportion of probing spent in E2 (%)	16	43.2 a ± 6.60	16	41.7 a ± 5.50	15	47.6 a ± 6.00	16.4	0.754

^1^ Number of replications; ^2^ only the EPG recordings that included a particular waveform were included in calculations; np—no probing (aphid stylets outside the plant tissues); C—pathway activity (extracellular stylet penetration with potential drops, i.e., short cell punctures); F—derailed stylet activities (difficulties in penetration); G—xylem phase (ingestion of xylem sap); E—phloem phase, including E1 (phloem salivation) and E2 (phloem sap ingestion); E2 > 10 min—sustained ingestion of phloem sap. Time and duration of various stylet activities are recorded in minutes. HSD_0.05_—honest significant difference at the 0.05 significance level. Different letters in rows show significant differences at *p* < 0.05 (ANOVA). Shading emphasizes variables that are significantly different between treatments.

**Table 2:** Probing behavior of *Myzus persicae* on *Brassica rapa* ssp. *pekinensis* untreated and treated with 0.1% apigenin and 0.1% luteolin: sequential EPG parameters.

	Control		Apigenin		Luteolin		HSD_0.05_	*F*- ANOVA
	n ^1^	Mean ± SEM	n ^1^	Mean ± SEM	n ^1^	Mean ± SEM		
Start of EPG								
Time to 1st probe from start of EPG	17	1.30 a ± 0.50	19	1.50 a ± 0.50	16	3.20 a ± 1.20	2.14	0.161
Duration of 1st probe	17	2.30 ab ± 0.70	19	0.70 b ± 0.30	16	3.70 a ± 1.50	2.67	0.048
Before 1st phloem phase								
Time from 1st probe to 1st E ^2^	17	183.1 a ± 30.5	19	199.6 a ± 35.7	16	170.9 a ± 29.3	92.1	0.820
Time to 1st E within the probe ^3^	16	35.9 a ± 9.00	16	19.8 a ± 2.60	16	25.4 a ± 4.20	16.2	0.131
Number of probes to 1st E1 ^2^	17	25.4 a ± 5.70	19	28.4 a ± 4.60	16	28.5 a ± 5.40	14.8	0.891
Duration of no probing before 1st E ^2^	17	37.9 a ± 12.6	19	44.8 a ± 9.20	16	32.0 a ± 7.50	28.5	0.665
1st phloem phase								
Duration of 1st phloem phase E ^3^	16	145.9 a ± 37.6	16	68.4 a ± 22.3	15	120.9 a ± 34.0	86.3	0.181
Before 1st sap ingestion phase E2								
Time from 1st probe to 1st E2 ^4^	17	190.3 a ± 30.5	19	213.6 a ± 35.3	16	171.7 a ± 29.3	91.6	0.654
Time to 1st E2 within the probe ^5^	16	37.6 a ± 8.90	16	20.00 b ± 2.60	15	26.2 ab ± 4.20	15.99	0.046
Before 1st sap ingestion phase E2 > 10 min								
Time from 1st probe to 1st E2 > 10 min ^6^	17	252.0 a ± 35.8	19	245.5 a ± 37.4	16	191.5 a ± 32.5	101.3	0.445
Time to 1st E2 > 10 min. within the probe ^7^	15	41.6 a ± 9.10	15	27.7 a ± 5.20	14	27.9 a ± 4.20	17.4	0.197
After 1st phloem phase								
Number of probes after 1st E ^7^	16	16.7 a ± 4.80	16	15.6 a ± 3.90	15	10.9 a ± 2.30	10.4	0.510
Number of probes < 3 min. after 1st E ^7^	16	11.4 a ± 3.80	16	10.3 a ± 2.70	15	6.40 a ± 1.70	7.80	0.430
Potential E2 index ^8^	15	64.8 a ± 9.00	15	58.6 a ± 7.70	14	63.1 a ± 7.90	22.3	0.84

^1^ Number of replications; ^2^ time or number of probes from 1st probe to the end of EPG recording if E is missing; ^3^ missing data if E is missing; E2; ^4^ time from 1st probe to the end of EPG recording if E is missing; ^5^ missing data if E is missing; ^6^ time from 1st probe to the end of EPG recording if E is missing; ^7^ missing data if E is missing; ^8^ potential E2 index = the percentage of time spent in E2 by an aphid with any sustained E2, after reaching the first sustained E2. Time and duration of various stylet activities are recorded in minutes. HSD_0.05_—honest significant difference at the 0.05 significance level. Different letters in rows show significant differences at *p* < 0.05 (ANOVA). Shading emphasizes variables that are significantly different between treatments.

## Data Availability

The data are provided in the present article.

## Supplementary Materials

The following supporting information can be downloaded at https://www.mdpi.com/article/10.3390/ijms26094452/s1.
