# Supplementary material for: Effects of Apigenin and Luteolin on Myzus persicae (Hemiptera: Aphididae) Probing Behavior
Source: Int J Mol Sci. 2025 May 7;26(9):4452. doi: 10.3390/ijms26094452 (PMC12072685; doi:10.3390/ijms26094452)
Supplement: Supplementary file 1 [file ijms-26-04452-s001.zip › ijms-3494397-supplementary.docx]

Table S1. Probing behavior of Myzus persicae on Brassica rapa ssp. pekinensis untreated and treated with 0.1% apigenin and 0.1% luteolin: non-sequential EPG parameters.

| **EPG variable** | **Control** | | | **Apigenin** | | | **Luteolin** | |  | **HSD_0.05_** | ***F*-ANOVA** |
| --- | --- | --- | --- | --- | --- | --- | --- | --- | --- | --- | --- |
|  | **n^1^** | **Min-Max** | **Median** | **n^1^** | **Min-Max** | **Median** | **n^1^** | **Min-Max** | **Median** |  |  |
| *No probing* | | | | | | | | | | | |
| Total duration of np | 17 | 11.2-221.9 | 39.3 | 19 | 10.2-135.1 | 75.8 | 16 | 7.7-129.3 | 47.8 | 31.5171 | 0.454 |
| Number of np | 17 | 12.0-100.0 | 27.0 | 19 | 11.0-85.0 | 37.0 | 16 | 12.0-74.0 | 32.5 | 16.48 | 0.936 |
| Mean duration of np | 17 | 0.7-2.2 | 1.4 | 19 | 0.8-3.6 | 1.5 | 16 | 0.4-2.4 | 1.3 | 0.4226 | 0.116 |
| *Probing* | | | | | | | | | | | |
| Total probing time | 17 | 258.0-468.8 | 440.7 | 19 | 345.0-469.8 | 404.2 | 16 | 350.7-472.0 | 432.2 | 31.5171 | 0.454 |
| Number of probes | 17 | 12.0-100.0 | 27.0 | 19 | 11.0-85.0 | 37.0 | 16 | 12.0-73.0 | 32.5 | 16.42 | 0.935 |
| Number of short probes (C<3 minutes) | 17 | 3.0-74.0 | 14.0 | 19 | 4.0-63.0 | 25.0 | 16 | 6.0-51.0 | 18.0 | 12.76 | 0.867 |
| *Pathway phase* | | | | | | | | | | | |
| Total duration of C | 17 | 52.4-357.4 | 182.4 | 19 | 93.0-360.6 | 196.5 | 16 | 68.6-307.9 | 197.0 | 56.8411 | 0.817 |
| Number of C | 17 | 13.0-100.0 | 33.0 | 19 | 17.0-88.0 | 41.0 | 16 | 15.0-78.0 | 34.0 | 16.52 | 0.927 |
| Mean duration of C | 17 | 1.3-13.5 | 4.8 | 19 | 1.8-9.7 | 4.7 | 16 | 3.3-8.7 | 5.1 | 1.7306 | 0.746 |
| Proportion of probing spent in C (%) | 17 | 11.6-100.0 | 43.9 | 19 | 20.5-100.0 | 51.4 | 16 | 14.7-78.8 | 45.5 | 16.6167 | 0.711 |
| *Derailed stylet activities* | | | | | | | | | | | |
| Total duration of F | 17 | 0.0-145.3 | 18.7 | 19 | 0.0-116.2 | 0.0 | 16 | 0.0-79.3 | 0.0 | 24.7516 | 0.623 |
| Number of F | 17 | 0.0-3.0 | 1.0 | 19 | 0.0-3.0 | 0.0 | 16 | 0.0-2.0 | 0.0 | 0.566 | 0.503 |
| Mean duration of F^2^ | 9 | 13.6-94.2 | 39.7 | 6 | 2.5-58.1 | 38.8 | 6 | 14.2-79.3 | 43.9 | 19.2192 | 0.376 |
| Proportion of probing spent in F^2^ (%) | 9 | 4.5-37.4 | 10.5 | 6 | 0.5-26.4 | 15.7 | 6 | 3.0-22.0 | 12.9 | 7.069 | 0.841 |
| *Xylem phase* | | | | | | | | | | | |
| Total duration of G | 17 | 0.0-55.1 | 0.0 | 19 | 0.0-79.9 | 4.7 | 16 | 0.0-67.2 | 0.62 | 16.3038 | 0.393 |
| Number of G | 17 | 0.0-3.0 | 0.0 | 19 | 0.0-2.0 | 1.0 | 16 | 0.0-2.0 | 0.5 | 0.531 | 0.977 |
| Mean duration of G^2^ | 8 | 15.0-29.6 | 21.3 | 10 | 4.7-75.5 | 37.0 | 8 | 1.2-67.2 | 18.8 | 13.619 | 0.04 |
| Proportion of probing spent in G^2^ (%) | 8 | 3.9-12.8 | 5.9 | 10 | 1.2-21.9 | 12.0 | 8 | 0.3-14.7 | 6.5 | 3.7032 | 0.044 |
| *Phloem phase: general* | | | | | | | | | | | |
| Total duration of phloem phase E (E1+E2) | 16 | 17.0-375.9 | 215.0 | 16 | 6.6-364.4 | 189.2 | 15 | 4.8-376.8 | 195.8 | 76.3053 | 0.752 |
| Total duration of E1 | 16 | 0.6-5.1 | 1.2 | 16 | 0.6-75.6 | 2.4 | 15 | 0.7-9.9 | 2.0 | 7.3755 | 0.252 |
| Total duration of E2 | 16 | 12.1-375.0 | 213.1 | 16 | 5.9-361.9 | 188.5 | 15 | 1.9-376.0 | 194.9 | 77.4006 | 0.693 |
| *Phloem phase: salivation (E1)* | | | | | | | | | | | |
| Number of E1 | 17 | 0.0-4.0 | 1.0 | 19 | 0.0-10.0 | 3.0 | 16 | 0.0-9.0 | 2.5 | 1.527 | 0.306 |
| Mean duration of E1^2^ | 16 | 0.6-1.7 | 0.76 | 16 | 0.6-25.0 | 0.8 | 15 | 0.5-1.6 | 0.76 | 2.4411 | 0.371 |
| Duration of the E1 followed by 1st E2^2^ | 16 | 0.6-5.0 | 1.2 | 16 | 0.6-7.2 | 2.4 | 15 | 0.7-5.4 | 1.9 | 1.0647 | 0.185 |
| Duration of the E1 followed by 1st E2 >10 min^2^ | 15 | 0.6-2.5 | 0.9 | 15 | 0.6-3.1 | 1.8 | 14 | 0.7-3.8 | 0.96 | 0.5951 | 0.035 |
| Contribution of E1 to phloem phase (%)^2^ | 16 | 0.2-29.4 | 1.0 | 16 | 0.2-58.5 | 1.8 | 15 | 0.2-66.2 | 0.9 | 9.0848 | 0.79 |
| Proportion of probing spent in E1 (%) | 16 | 0.1-1.4 | 0.3 | 16 | 0.2-19.2 | 0.7 | 15 | 0.1-2.4 | 0.57 | 1.8788 | 0.261 |
| *Phloem phase: sap ingestion (E2)* | | | | | | | | | | | |
| Number of E2 | 17 | 0.0-4.0 | 1.0 | 19 | 0.0-10.0 | 3.0 | 16 | 0.0-8.0 | 2.0 | 1.445 | 0.39 |
| Number of E2 >10 min | 17 | 0.0-3.0 | 1.0 | 19 | 0.0-4.0 | 1.0 | 16 | 0.0-5.0 | 1.0 | 0.82 | 0.253 |
| Mean duration of E2^2^ | 16 | 4.1-375.2 | 71.2 | 16 | 5.9-304.3 | 45.4 | 15 | 0.9-376.0 | 117.2 | 80.9156 | 0.195 |
| Proportion of probing spent in E2 (%) | 16 | 3.4-80.6 | 47.2 | 16 | 1.7-78.8 | 46.8 | 15 | 0.5-80.6 | 47.0 | 16.4335 | 0.754 |

^1^ Number of replications; ^2^ only the EPG recordings that included a particular waveform were included in calculations; np—no probing (aphid stylets outside the plant tissues); C—pathway activity (extracellular stylet penetration with potential drops, i.e., short cell punctures); F—derailed stylet activities (difficulties in penetration); G—xylem phase (ingestion of xylem sap); E—phloem phase including E1 (phloem salivation) and E2 (phloem sap ingestion); E2 > 10 min—sustained ingestion of phloem sap. Time and duration of various stylet activities are given in minutes. HSD_0.05_—honest significant difference at the 0.05 significance level. Different letters in rows show significant differences at *p*<0.05 (ANOVA). Shading emphasizes variables that are significantly different between treatments.

Table S2. Probing behavior of *Myzus persicae* on *Brassica rapa* ssp. *pekinensis* untreated and treated with 0.1% apigenin and 0.1% luteolin: sequential EPG parameters.

|  | **Control** | | | **Apigenin** | | | **Luteolin** | | | **HSD_0.05_** | ***F*-ANOVA** |  |
| --- | --- | --- | --- | --- | --- | --- | --- | --- | --- | --- | --- | --- |
|  | **n^1^** | **Min-Max** | **Median** | **n^1^** | **Min-Max** | **Median** | **n^1^** | **Min-Max** | **Median** |  |  |  |
| *Start of EPG* | | | | | | | | | | | | |
| Time to 1st probe from start of EPG | 17 | 0.0-7.3 | 0.6 | 19 | 0.0-9.2 | 0.5 | 16 | 0.02-18.4 | 1.1 | 2.1438 | 0.161 |  |
| Duration of 1st probe | 17 | 0.5-9.2 | 1.2 | 19 | 0.1-4.1 | 0.32 | 16 | 0.2-22.2 | 0.6 | 2.6658 | 0.048 |  |
| *Before 1^st^ phloem phase* | | | | | | | | | | | | |
| Time from 1st probe to 1st E^2^ | 17 | 24.5-479.9 | 156.8 | 19 | 33.4-480.0 | 123.1 | 16 | 29.1-475.0 | 142.3 | 92.1198 | 0.82 |  |
| Time to 1st E within the probe^3^ | 16 | 7.1-149.3 | 26.4 | 16 | 8.0-43.5 | 15.1 | 15 | 7.8-63.3 | 18.3 | 16.1789 | 0.131 |  |
| Number of probes to 1st E1^2^ | 17 | 2.0-100.0 | 17.0 | 19 | 6.0-59.0 | 21.0 | 16 | 3.0-68.0 | 21.0 | 14.79 | 0.891 |  |
| Duration of no probing before 1st E^2^ | 17 | 5.6-221.9 | 25.5 | 19 | 6.6-134.7 | 23.6 | 16 | 1.8-94.3 | 20.9 | 28.5167 | 0.665 |  |
| *1^st^ phloem phase* | | | | | | | | | | | | |
| Duration of 1^st^ phloem phase E^3^ | 16 | 1.0-375.9 | 69.4 | 16 | 0.7-305.0 | 21.9 | 15 | 1.1-376.8 | 60.4 | 86.3244 | 0.181 |  |
| *Before 1^st^ sap ingestion phase E2* | | | | | | | | | | | | |
| Time from 1st probe to 1st E2^4^ | 17 | 25.1-479.9 | 175.4 | 19 | 34.5-480.0 | 147.1 | 16 | 29.9-475.0 | 143.1 | 91.6255 | 0.654 |  |
| Time to 1st E2 within the probe^5^ | 16 | 7.5-150.0 | 28.0 | 16 | 9.1-44.2 | 16.0 | 15 | 8.6-64.0 | 19.0 | 15.9995 | 0.046 |  |
| *Before 1^st^ sap ingestion phase E2>10 min* | | | | | | | | | | | | |
| Time from 1st probe to 1st E2>10 min^6^ | 17 | 25.1-479.9 | 243.5 | 19 | 56.0-480.0 | 194.6 | 16 | 57.1-475.0 | 157.1 | 101.324 | 0.445 |  |
| Time to 1st E2> 10 min. within the probe^7^ | 15 | 12.9-150.0 | 29.4 | 15 | 13.0-91.5 | 17.8 | 14 | 14.7-64.0 | 21.3 | 17.3832 | 0.197 |  |
| *After 1^st^ phloem phase* | | | | | | | | | | | | |
| Number of probes after 1st E^7^ | 16 | 0.0-56.0 | 12.5 | 16 | 0.0-47.0 | 11.5 | 15 | 0.0-27.0 | 11.0 | 10.41 | 0.51 |  |
| Number of probes < 3 min. after 1st E^7^ | 16 | 0.0-48.0 | 4.5 | 16 | 0.0-33.0 | 8.5 | 15 | 0.0-23.0 | 5.0 | 7.8 | 0.43 |  |
| Potential E2 index^8^ | 16 | 3.8-100.0 | 66.0 | 16 | 1.7-100.0 | 65.5 | 15 | 1.1-100.0 | 61.6 | 22.2988 | 0.84 |  |

^1^ Number of replications; ^2^ time or number of probes from 1st probe to the end of EPG recording if E is missing; ^3^ missing data if E is missing; E2; ^4^ time from 1st probe to the end of EPG recording if E is missing; ^5^ missing data if E is missing; ^6^ time from 1st probe to the end of EPG recording if E is missing; ^7^ missing data if E is missing; ^8^ potential E2 index = the percentage of time spent in E2 by an aphid with any sustained E2, after reaching the first sustained E2. Time and duration of various stylet activities are given in minutes. HSD_0.05_—honest significant difference at the 0.05 significance level. Different letters in rows show significant differences at *p*<0.05 (ANOVA). Shading emphasizes variables that are significantly different between treatments.
